# Integration between Primary Health Care and Emergency Services in Brazil: Barriers and Facilitators

**DOI:** 10.5334/ijic.4066

**Published:** 2018-11-15

**Authors:** Liza Yurie Teruya Uchimura, Andréa Tenório Correia da Silva, Ana Luiza d’Ávila Viana

**Affiliations:** 1 Department of Preventive Medicine, Faculty of Medicine, University of Sao Paulo, BR; 2 Faculty of Medicine Santa Marcelina, BR

**Keywords:** integrated care, primary health care, emergency care, systems integration, health systems, health policy

## Abstract

**Introduction:**

Characteristics of primary health care and emergency services may hamper their integration and, therefore, reduce the quality of care and the effectiveness of health systems. This study aims to identify and analyse policy, structural and organizational aspects of healthcare services that may affect the integration between primary health and emergency care networks.

**Theory and Methods:**

We conducted a qualitative research study based on grounded theory that included: (1) interviews with 30 health care leaders; and (2) documental analysis of the summaries of Regional Interagency Committee meetings from two regions in the state of Sao Paulo, Brazil.

**Results:**

The integration between primary health and emergency care network is inefficient. The barriers that contributed to this situation are as follows: (1) policy: the municipal health department is responsible for providing primary health care and the regional health department provides emergency care, but there is a lack of space for the integration of services; (2) structural: distinct criteria for planning mechanisms; and (3) organizational: ineffective point of interaction between different levels of the health system.

**Conclusions and discussion:**

Our findings have implications for health management and planning in low-and middle-income countries (LMICs) with suggestions for interventions for overcoming the aforementioned barriers.

## Introduction, comprising background and problem statement

Integration is the cornerstone of effective health systems with a coherent set of methods and models to connect, align and collaborate within and between health sectors [1,2], since it enhances the quality of care, and patient safety as well as helping to reduce healthcare system costs [3]. Evidence shows that integrated care can also reduce hospitalization, demand for emergency department and average length of stay [4]. Integrated care has four dimensions (professional, organizational, functional and normative integration) [5] and it is rooted in networks such as organizational structure for the production of services and organizations [6]. The increased discussion about integrated care was caused by the aging population and complications associated with chronic diseases; the rising complexity of skills to provide integral care to patients, and an increase in the specializations from health professions with a consequent fragmentation of care [7].

Integration is particularly important for countries with resource-constrained health systems, such as several low- and middle- income countries, and regions where health inequalities are on the rise. The implementation of regionalized health systems, to overcome the divergence between situational diagnosis (increase of chronic diseases) and the current health actions (directed toward acute pathologies) has led to the creation of the Health Care Network in Brazil [8]. The Health Care Network has been defined as a set of actions and health services, connected through technical, logistical and management support systems [9]. The creation of a Health Care Network with shared responsibility in health care, intersectoral actions and approach on health determinants is expected to improve integration between primary health care and emergency care network.

Studies have shown that the more integrated health systems are, the greater care coordination and hospital effectiveness is [10]. For instance, in Brazil there was evidence of inefficient integration between emergency services and primary health care. In 2011, Brazil implemented walk-in clinics to improve integration and relieve the growing demand for emergency services by reformulating the National Emergency Care Policy to create the emergency care network [11,12]. The document defined the guidelines for the network, its hierarchical framework, and described components and objectives to be achieved through service regionalization [11]. Evidence shows that the different services that make up the emergency network, walk-in clinics in the state of Parana [13] and Rio de Janeiro [14] and the mobile emergency medical service (SAMU) [15] are not integrated into the Health Care Network.

Nowadays, the interfaces that integrate primary health care and the emergency network in Brazil are limited to the referral of patients between the two levels of the health system, when it exists, although it is not mandatory, and it is up to the health professional to perform this interface. The lack of patient care coordination, along with the absence of a protagonist for its integration into the Brazilian health system, and the difficulties of the population in passing through the health services show how the interface between primary care and emergency services presents a problem faced by managers and providers. The lack of articulation between the different actors of the health system in Brazil makes it difficult to plan for integrated care based on the health needs of the population, as well as to follow the current health policy. However, a few studies have addressed the integration between primary health care and emergency services in low- and middle- income countries.

Our study examines the integration between the primary health care and the emergency care network in Brazil. We aimed to answer the following questions: (1) Do policy, structural and organizational aspects compromise the integration of primary health care and the emergency care network in Brazil? (2) Do the primary health and emergency care network work properly? Answering these questions and identifying characteristics of health services that affect the integration of these two levels of health care may help provide guidance for the creation of interventions which enhance the quality of care and, consequently, improve healthcare indicators, care management, user satisfaction, and the cost-effectiveness of health systems.

## Theory and Methods

### Study design and participants

We performed a qualitative study, based on grounded theory, with two sources of information: (1) interviews with closed-ended and open-ended questions, and (2) the evaluation of the minutes of Regional Interagency Committee meetings in North-Barretos and South-Barretos from January to August 2015, the months in which the interviews took place in the regions, totalling 15 documents.

### Study location

The Regional Health Department-V covers 8,099 km^2^ and is divided into two regions: North-Barretos and South-Barretos, with 18 cities. These regions were selected as they represent the urban complexity; socioeconomic diversity; the predominant health care provider profile; and the diverse health system situations throughout different regions of Brazil.

Also used as a selection criterion was the typology of the health regions proposed by Viana et al. [16] (North-Barretos is group 5 – high socioeconomic development and high offer of services – and South-Barretos is group 3 – average socioeconomic development and mid/high range of services). The selected municipalities were those that reported the largest numbers of health facilities in North-Barretos and South-Barretos (Olimpia and Bebedouro, respectively), the main city of Regional Health Department-V (Barretos) and those with smaller numbers of health services in significantly populous regions (Cajobi and Taiuva). This study is part of the research “Policy, Planning and Management of Health Care Regions and Networks in Brazil” [17].

The study regions are in the state of Sao Paulo, where socioeconomic indicators are higher than the national average. The main health condition rates in the regions are highlighted in Table 1.

**Table 1:** Rates of health conditions for North-Barretos, South-Barretos, Sao Paulo and Brazil, 2015.

Rates	North-Barretos	South-Barretos	State of Sao Paulo	Brazil

Life expectancy at birth	74.9	75.02	75.69	73.40
Child mortality	9.78	12.04	11.58	13.51
Mortality due to ischemic heart diseases (100,000 inhab.)	63.96	46.23	67.36	54.08
Mortality due to cerebrovascular diseases (100,000 inhab.)	66.04	62.06	51.61	51.73
Cancer mortality (100,000 inhab.)	127.91	128.06	116.33	98.47

Source: Região and Redes, 2013.

Life expectancy at birth in the study regions is higher than the national average. Child mortality rates are lower in the study regions compared to the state of Sao Paulo and national averages, with the index for North-Barretos being the highlight. Elevated rates for conditions linked to chronic diseases, such as mortality from ischemic heart and cerebrovascular diseases, suggest insufficient primary health care. The high mortality rates for neoplasms relate to the presence of the Barretos Cancer Hospital, a nationally renowned cancer treatment service, managed by the Pio XII Foundation.

### Participants and Data collection

The health leaders were identified, including managers, service providers and community representatives, working in primary health care, (advisors, doctors, managers from primary care centres) emergency care networks (directors and doctors from emergency departments; specialized centres, 24 h emergency care clinics, emergency mobile care services) or the management of health care levels in the context of the state, regional and municipal spheres of government (directors from emergency care networks, primary care, hospitals, regulation; Municipal Health Department directors and Regional Health Department directors). They were selected according to the basic tenet of grounded theory: participants who were believed can offer valuable insight into the issue under research [18]. Initially we interviewed two managers, and regarding the information gathered from theses interviews we selected further participants. We invited to participate healthcare managers who were working in administrative position at the time of the data collection.

The 28 health leaders were contacted by telephone and/or e-mail to arrange the schedule. The interviews were held in August 2015 at each interviewee’s workplace. The health leaders were presented with the questionnaires, which consisted of questions about primary health care and emergency care networks and were conducted and completed by the researcher. The questions for all respondents covered policy, structural and organizational aspects of health systems.

For the policy aspect, we tried to identify the areas of action, negotiation and conflict in the region, the processes, decision flows, and the conduct of the policy and functions exercised by each institution in health decisions in the region. The structural aspect reflects the capacity of health services, monitoring and evaluation of health services, availability and sufficiency of physical, financial and human resources. The organizational aspect presents the criteria for conformation of Health Care Network, planning, management, systemic integration between services, regulation, and access of the population to health care services.

The questionnaire for two state managers featured open-ended questions and the interview followed these questions. The choice of this script for the central level managers of the state is due to the better understanding and maximum depth of the interviewee’s different points of view, respecting the principle of the free association of ideas while conducting the interview. These interviews were transcribed. The material was read out repeatedly, followed by thematic content analyses, prioritization of the issues and the establishment of relationships between them. The analysis was performed by two more researchers to ensure reliability.

We chose to analyse the minutes of Regional Interagency Committee meetings from January to August 2015 to contextualize discussions held in previous meetings until the time of the interviews with the managers in these regions. The resulting documents reflected the participation of representatives from the 17 municipalities of the region, except for one from South-Barretos, who failed to partake in any of the meetings. Municipal health secretaries, except in the case of the municipality-hub, attended all the meetings or sent representatives on their behalf. The documents were read repeatedly to present the topics of analysis and thus generate the data.

### Data analysis and ethical aspects

The data were tabulated using the open source PHP software – LimeSurvey. Statistical computations were performed using SPSS Statistics for Windows, Version 22.0 (Armonk, NY: IBM Corp). The descriptive statistical results were presented as the median scores regarding each of the issues, expressed on the five-point Likert scale, where one (1) corresponded to the worst rating and five (5) to the best. Average scores of equal to or greater than three (3) indicated a positive evaluation.

We used the grounded theory as the qualitative research method since it enables researchers to capture and understand health care experiences, it is focused toward building theory and is a method commonly used in health research [18]. Grounded theory has approach to qualitative data an inductive method of developing new theory based on the possibility of interpretation from specific phenomenon to general and it is openness to multiple explanations in the process of generation of qualitative theory [19,20]. In grounded theory it is possible the continuous categorization comparing with the literature and provides an analysis with a generation of theory, this being one of the characteristics that allowed to be so used in health research [20]. The use of grounded theory is also justified by the pre-existing knowledge about the problem with the simultaneous process of data collection with the analysis, development of categories and codes from the data collected.

Thematic content analysis was conducted using Atlas-ti software and the theme categories were generated representing the following aspects: policy, structure and organization. The thematic analysis was undertaken which followed the principles of grounded theory with learning from the data and not from an existing theoretical vision [21,22].

The study was approved by the Ethics Committee of the University of Sao Paulo School of Medicine, with case reference number 045/16, and in accordance with National Health Council rule number 466/12.

## Results

**Figure 1:**
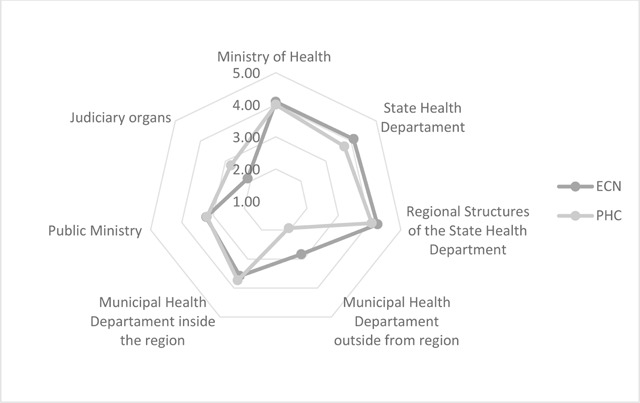
Main institution responsible for the decision-making in primary health care (PHC) and the emergency care network (ECN), North-Barretos and South-Barretos regions, 2015. Source: Prepared by the authors.

Most of the respondents answered that the main institution responsible for the organization of the emergency care network in the study regions was the Regional Health Department of the State Health Department (Figure 1) also in terms of decision-making at emergency care. On the other hand, the main institution responsible for the decision-making in primary care in these regions was the Municipal Health Department (Figure 1).

Regional Interagency Committee and emergency care group advisors were reported as relevant for decisions on emergency care network. Conflicts in decision-making on the emergency care network occurred between the municipalities of the region and between the municipalities and the regional structure. However, according to the State Health Department the Regional Interagency Committee has become a space with little decision-making power:

“…it has become much more of a space to meet the agenda than deliberations, not involving a good part of the important people from the municipalities, so at some Regional Interagency Committees you have someone from the municipality who is going to meet the schedule but not comply with the role of deliberation…”

Regional Interagency Committees, National Councils of Municipal Health Departments and Health Councils were defined as relevant spaces for decisions in primary health care. Conflicts in decision-making concerning primary health care occurred between the municipalities of the region and the Regional Health Department, between public managers and health professionals and between specialist doctors and other health professionals.

Summaries from the Regional Interagency Committee of North-Barretos demonstrated that the emergency care network was discussed more in comparison to primary health care. The latter had not been discussed in relation to service coverage but rather regarding professional training in exams like the red reflex test. At the Regional Interagency Committee of South-Barretos, primary health care was discussed more compared to the emergency care network, and most of the themes were about the organization and professional training.

The planning of the emergency care network considered the regional service provision plan, as well as medical and therapeutic support. According to the interviewees, the most commonly used criteria for this planning were the diagnosis of health and the installed capacity of health services. The State Manager added that the organization of this network had different ways of planning and organization between the North-Barretos and South-Barretos regions due to the pre-existing services and the financing of each one:

“… the emergency care network did not have this character to emerge even from the municipalities. They already have several structures. North-Barretos already has SAMU and some municipalities already have walk-in clinics; The Santa Casa Hospital in Barretos has always been highly regarded for emergency care. South-Barretos has not; … is very much – despite being a region of rich municipalities in terms of health equipment, they are precarious, they have nothing of high complexity in the territory, do not do any high procedure.”

Only 44% of the health leaders identified formal coordination for the emergency care network in these regions, composed of the State Manager, Municipal Managers and health providers. Moreover, there is little definition of the roles and functions of federal and state managers and even less so for the municipal ones. For the State Manager, the presence of a well renowned and important health provider in this region demonstrates how this Foundation is important for the organization of regional services:

“…the Pio XII Foundation in the region of Barretos is admittedly an institution of national importance, not only regional, not only state, but of national importance….”

The organization of primary care contemplated the diagnosis of health care needs, the Regional Plan for service provision and diagnostic and therapeutic support. Health leaders agreed that primary care has guaranteed prompt care for the people who use these services. The analysis of the minutes from the Regional Interagency Committee meetings demonstrated a scenario with a deficiency of primary health care, in relation to the appropriation of the population’s health needs, and primary care for cases of suspected or diagnosed dengue, with difficulties to control the disease vector and promote appropriate care. Important differences and similarities were identified between primary health care and the emergency care network in the regions studied; below we show the main themes (Table 2).

**Table 2:** Differences and similarities between primary health care and the emergency care network in the North-Barretos and South-Barretos regions, 2015.

	Primary Health Care	Emergency Care Network

**Services**	Primary Care Centre, Family Health Support Centre (NASF), Outpatient medical specialties	Emergency Departments, Walk-in clinics
**Interfaces**	Primary Care Centre	Walk-in clinics
**Institution responsible for organization**	Municipal Health Department	Regional Health Department
**Space for decisions**	Municipal Health Department	Regional Health Department
**Conflicts for decision making**	Municipalities vs. Municipalities	Municipalities vs. Regional Health Department
**Planning**	Diagnosis of health needs	Regional Plan for the provision of services

Source: Prepared by the authors.

According to 58% of the interviewees, there was integration of primary health care with the emergency care network. However, only 19% answered that there was a referral and counter-referral mechanism from the emergency services to primary health care. Despite this, 62.5% of respondents stated that there was integration with emergency services and that there had been improvements in the health system to manage this network. The definition of care flows is the emergency care network’s main contribution to these regions. However, no document, between January and August 2015, contained any reference to integration between these two levels.

Emergency regulation addresses the need for access and the demand for beds and contributes to the planning and organization of the network. This operated at state level for 90% of the respondents, and with well-defined protocols and flows for 80% of the respondents. The main themes discussed at the Regional Interagency Committee about emergency care network formations were the geographical coverage of health services (77%), health service providers (66%), and regulation (66%). The regulation cited in the summaries is related to the redefinition of the quotas of each municipality for referral to the reference hospitals of the municipality hub for exams and flows of authorizations for hospital admission in the region.

Emergency providers in North-Barretos and South-Barretos can be evaluated based on their production target (25%), productivity (25%), results (25%) and quality target (12.5%). The services are evaluated through performance indicators. However, the results of monitoring and evaluation are only used by a few managers in the network planning (average score: 2.1). The monitoring and evaluation process is carried out for municipal managers (78%), state managers (67%), Regional Interagency Committee coordinators (67%), regional emergency coordinators (55%) and state emergency coordinators (44%). This process involves little participation by regional, state and municipal regulation coordinators and by the state health council.

Primary health care teams from North-Barretos and South-Barretos were responsible for the main activities: prenatal consultations and cytopathological examination. In summary, the most cited activities related to primary health care included: training for physicians to improve the diagnosis of melanomas and mental health diseases; oral cancer training for dentists; and the recruitment of new community health agents. Despite these activities, the State Manager stated that primary care is not fulfilling its role as a coordinator of care and most of emergency service appointments could be performed in the primary health care services:

“…oversized emergency service structures reinforce a distortion of the model, the role of primary health care is not being fulfilled, creating a high demand for the walk-in clinics and emergency departments.”

The funding of primary health care and the emergency care network in the study regions shared the same features. Municipal coffers provided the bulk of the funding, followed by federal resources and a smaller contribution from the state level. State managers agreed that there had been no improvements in these two levels of the health system.

According to 64% of respondents, primary care subjects discussed by the Regional Interagency Committee are related to the coverage of services, funding, and coordination with the network. Among the interviewees, 58% referred to the comprehensiveness of care, 53% to human resources, and 47% to the scope of action at this level of care. Summary documents reflected a different view; there were more reports of pacts between emergency services and their funding. The minutes from Regional Interagency Committee demonstrated that primary health care had not been discussed in relation to service coverage, but rather regarding professional training, and most of the themes were about the organization and professional training.

The data collected in the interviews and the analysis of the summaries allowed us to identify facilitators and barriers for the integration of health care between primary care and emergency services (Table 3). We consider factors that facilitate the integration of care into important tools for strengthening the health system.

**Table 3:** Facilitators and barriers to the integration of primary health care and the emergency care network by policy, structure and organization aspects, 2015.

Integration between primary health care and emergency care network	Facilitators	Barriers

**Policy aspect**	Regional Interagency Committee	Lack of integration between the municipal health department and the regional health department in order to provide comprehensive care
**Structure aspect**	Sufficient funding	Distinct criteria for planning mechanisms
**Organization aspect**	Professional training (Pio XII Foundation)	Ineffective interfaces: there is no integration between services

Source: Prepared by the authors.

## Discussion

Through our data sources, we suggest a grounded theory for the integration of primary care with emergency services in Brazil based on the identification of policy, structural and organizational aspects. We observed in our study the challenges of integration with limitations for total consolidation. In the policy aspect the main difficulty identified is in the different institutions that organize these two levels of attention. In the structural aspect the incompatibility of planning mechanisms and in the organizational aspect the uncommon points of interfaces in the health system. On the other hand, the identification of facilitators for this integration according to the perception of several data sources highlights the importance of the study for regional policy and to integrate care.

The organization of primary health care by the Municipal Health Department and the emergency care network by the Regional Health Department demonstrates difficulties for the same institution to maintain this planning. Other studies in these regions [23,24] have confirmed the regional institution as the main service organizer in this area.

Our results have shown that planning for primary health care and the emergency care network is incipient and fragile in relation to the health needs of the population. There is also evidence of minimal monitoring and evaluation of health indicators. One successful experiment that built this model, centred on patients and health care needs, was a network of researchers, clinicians, and managers. The union of academic and clinical activities helped in planning the integration of primary health care and emergency care networks [25].

The integration of interfaces in primary health care with the emergency care network is limited to referral and counter-referral mechanisms, and in most cases is dependent on the health professional, rather than being a standardized activity in the system. While making therapeutic itineraries with stroke patients in the same regions, Bousquat et al. [23] reported similar results with no communication between services and professionals at different levels of assistance. They confirmed through interviews with patients in this clinical state that there are no mechanisms of continuity of care and care flow or vertical integration.

The recognition of different interfaces highlights the problems of access to health care and non-continuity of care. An example of this is when patients receive care at walk-in clinics and are not referred for follow-up at the primary care centre. In case studies by Almeida et al. [26], in municipalities in Brazil and Spain, the counter-referral grounds were also demonstrated and justified according to the patient’s preference in relation to specialized care, isolation between professionals of the two levels of care, underqualification of the primary care physician and the difficulties experienced by professionals in recording clinical data.

Our study identified that the largest contribution made by the emergency care network was the definition of flows, even with partial integration between network components. Planning the integration of interfaces from health system services involves the responsibility of different actors involved and considers geographical factors, historical relevance and organizations leadership. Brown and Oliver-Baxter [5] suggest this planning, based on dimensions, to obtain an optimal integration of the health system. The professional dimension should carry out different partnerships among health professionals and thus align their competencies, responsibilities and knowledge. The organizational dimension suggests that there is a sharing of governance mechanisms to define the best care for the population. The functional dimension aims to support the financing and health information system. Finally, the normative dimension is aimed at common goals among organizations and health professionals in the health system. Topp and colleagues [27] have identified that the integration of health systems in low- and middle-income countries relies on functional health services, trained and motivated health professionals, the availability of appropriate tools to enhance the integration of services, and processes which are flexible according to local circumstances.

For Harrop [28] the information systems connected to the emergency care network and primary health care can help to build a strategy that moulds the network’s operation, aimed at a variety of common objectives. Evidence has proven that there is a minimum level of coordination between emergency care services and primary care, even in systems with electronic patient records interconnecting both levels of care. The interfaces to integration should be operated as a whole, from the human resources to the clinical protocols [29] and, according to Patel and Kushniruk [30] the term ‘interface’ in health care often refers simply to an information system of effective patient records.

Asante and colleagues [31] point out that in recent years primary health care has benefited mainly the poorest populations, while the hospital services have benefited the better-off in low-and middle-income countries of sub-Saharan Africa, the Asia-Pacific region, Latin America and the Middle East. They concluded that health care financing in LMICs favoured people with better socioeconomic levels even with investments in primary care to increase equity. In addition, other authors report that most primary care programs in LMICs have components, such as improved access, and effective and long-term care, for a financial reform based on the health needs of their population [32].

The identification and development of spaces for the governance of health services, whether national or sub-national, are important mechanisms for the integration of health systems in LMICs [29]. Regional Interagency Committee meetings should be this space in North-Barretos and South-Barretos, however, they proved to be more of a deliberative space with few discussions. In addition, there were some disagreements among the interviewees with the reading of summaries. Other studies in different regions of Brazil revealed the same characteristics as the Regional Interagency Committee which we analysed [33,34], however, this forum acts as an important indicator of the effectiveness of regionalization [35,36].

Vargas et al. [37] affirmed that any effective implementation of Health Care Network in Brazil is dependent on negotiation, the division of responsibilities among managers, and on an increasing participation at state and federal levels; suggesting a strengthening of the regional structure. Brown et al. [38] report dissatisfaction among various system components with the evaluation of the patient results and with their service providers’ responses in the health systems. It has been found that regionalization is not a health policy or process that is sufficient for the integration of optimal care and services, nor for efficient coordination and regulation. Viana et al. [39] affirmed that regionalization is a new culture for the provision of health care, with improvements in the availability of health services and actions in order to provide integrated care with good use of resources and economically.

## Conclusion

Policy, structural and organizational aspects influenced the integration between primary health care and emergency care networks. We found that the barriers for the integration between primary health care and the emergency care network were: (1) the municipal health department being responsible for providing primary health care and the regional health department being responsible for providing emergency care, however there is lack of space for the integration of services; (2) distinct criteria for planning mechanisms – diagnosis of health needs are used in primary health care and Regional Plan for the provision of services to the emergency care network; and (3) ineffective interfaces between different levels of the health system, meaning there is no integration and no continued care. We identified that, the policy of regionalization with health care networks is not aligned with the needs of the population or the capacity for integration and coordination of care at different levels of the health system.

We suggest the following interventions to overcome the aforementioned barriers: (1) improving the coverage of primary health care may improve the integration between primary care and the emergency network; (2) binding responsibilities of integration between primary care managers and emergency services; (3) health planning focused on the health needs of the population at both levels of the health-system; (4) well-defined interfaces and services between primary care and emergency services; (5) actor responsible for the integration of care at different levels of health care, and health professionals able to work with these interfaces. For future health policies in Brazil, it is necessary to include recommendations about dealing with these challenges in the National Primary Health Care Policy and the National Emergency Care Policy.

Healthcare managers should integrate the different health services and share knowledge of population health diagnosis. The fragmentation of health management is reflected in the fractionation of health care. We believe that in order to achieve efficient integration among healthcare services, stakeholders and policy makers should prioritize standardized rules of high performance management, teamwork forums, leadership training courses and programs for monitoring each dimension of integration (professional, organizational, functional and normative integration).
